# Properties of Human Gastric Lipase Produced by Plant Roots

**DOI:** 10.3390/life12081249

**Published:** 2022-08-16

**Authors:** François Guerineau

**Affiliations:** BioEcoAgro Research Unit, Université de Picardie Jules Verne, 33 Rue St Leu, 80039 Amiens, France; francois.guerineau@u-picardie.fr

**Keywords:** *Arabidopsis*, hairy roots, lipase, protein production

## Abstract

The properties of recombinant human gastric lipase produced in *Arabidopsis thaliana* roots have been investigated with the goal of determining the potential of the enzyme. This enzyme is stably bound to roots and can be extracted using a buffer at pH 2.2. This enzyme retains over 75% of its activity after two weeks at room temperature when stored in a pH 2.2 buffer. Some of this activity loss was due to the adsorption of the enzyme to the surface of the container. There was no loss of lipase activity in dehydrated roots stored at room temperature for 27 months. The half-life of the enzyme was approximately 15 min when stored in solution at 60 °C whereas dried roots retained 90% lipase activity after one hour at 80 °C. In vitro binding assays using different root cell wall extracts suggested that the lipase was bound to pectin in the roots. Lipase released from the root powder hydrolyzed tributyrin. The high stability of the recombinant human gastric lipase makes this enzyme a good candidate to be tested as a catalyst, whether in solution or bound to roots.

## 1. Introduction

Lipases (EC 3.1.1.3) are enzymes of the hydrolase family that: (i) act on triacyl glycerides as part of the catabolic process required for fatty acid absorption and (ii) exhibit esterase activity. The two main lipases involved in digestion in humans are produced by pancreas and stomach cells [1]. Unlike pancreatic lipase, gastric lipase (GL) acts at acidic pH and does not require a colipase for activity [2]. Gastric lipase is a soluble enzyme found at the interface of lipid droplets and aqueous solutions [3]. The hydrophobic active center inside the enzyme structure is covered by a lid that opens upon contact of the enzyme with a substrate [4].

The low pH tolerance of gastric lipase makes it a candidate for enzyme supplementation therapies for pancreatitis or cystic fibrosis patients [5]. Lipase supplementation is currently done using enzymes extracted from livestock. A human recombinant enzyme would be an interesting alternative to lipases of animal origin [6]. Lipases, especially of microbial origin, have been used as biocatalysts in numerous biosynthetic reactions [7]. They can act in various solvents or ionic liquids to produce innovative molecules of therapeutic interest [8]. Human gastric lipase may also be used as a catalyst in esterification or transesterification reactions.

Plants are now considered valuable hosts for the production of heterologous proteins [9]. As a consequence of the ban on transgenic crops in some parts of the world, in vitro plant systems have been developed for the production of heterologous proteins with the first approved plant-produced protein for therapeutic use being glucocerebrosidase, produced by transgenic carrot cells grown in bioreactors [10]. Hairy roots are another in vitro system that can be used for the production of heterologous proteins [11]. Hairy roots emerge following infection of plant tissue by the bacteria *Rhizobium rhizogenes*. Hairy roots have higher stability than alternative cell culture systems, likely because they can grow without the need to add phytohormones which can induce genetic instability [12,13,14]. The Brassicaceae plant family can be used as an alternative to tobacco species for the production of heterologous proteins [15,16,17]. In particular, *Arabidopsis thaliana* hairy roots have been established for the production of human gastric lipase [18]. Although the enzyme, in this system, was targeted for secretion, it did not diffuse from the hairy roots to the culture medium and had to be extracted from the roots, which suggested some interactions between the enzyme and the roots producing it. In this work, I further investigated the interaction of gastric lipase with the roots. Given that the stability of an enzyme produced in a heterologous host was sometimes found to be lower than that of the native enzyme [19], I investigated if this was similarly the case in recombinant human gastric lipase produced by *Arabidopsis thaliana* hairy roots. This investigation paves the way for using this recombinant human gastric lipase enzyme as a biocatalyst or for lipase supplementation in certain patients.

## 2. Materials and Methods

### 2.1. Materials

The *Arabidopsis thaliana* hairy root line used in this work was the GL28 line described in [18]. It was obtained by hypocotyl transformation of the *Arabidopsis sgs3-12* co-suppression mutant line with the *Rhizobium rhizogenes* 15834 strain containing the cDNA encoding the mature human gastric lipase, fused to a plant signal peptide-encoding sequence, under control of the 2 × 35 S cauliflower mosaic virus promoter. The lipase substrates 4-methylumbelliferyloleate (MUO) (ref. 75164) and tributyrin (ref. W222305) were from Sigma-Aldrich, St-Quentin-Fallavier, France.

### 2.2. Root Culture and Storage

Hairy roots were grown at 21 °C on an orbital shaker at 60 RPM in 55 mm diameter Petri dishes in Gamborg B5 medium containing sucrose at 5% and 2,4-D at 0.5 mg/L. They were harvested after 18 to 21 days, briefly washed in distilled water, and freeze-dried overnight. The dehydrated roots were kept in Eppendorf tubes having their lids punctured. The tubes were placed in closed containers containing silica gel and stored in the dark at room temperature.

### 2.3. Lipase Extraction and Assay

Lipase was extracted by grinding 5 to 10 mg of dry roots in 200 to 400 µL of lipase extraction buffer (LEB: 0.1 M glycine-NaOH pH 2.2, 0.15 M NaCl). The mixture was incubated for 15 min at 37 °C in an Eppendorf Thermomixer at 1000 RPM and then centrifuged at 13,000× *g* for 2 min. Fluorometric assays were done on the supernatants diluted 1/100 or 1/200 in LEB in Eppendorf Protein LoBind^®^ tubes. As previous work has established linearity of lipase activity on tributyrin and esterase activity on MUO [18], for convenience, the fluorometric assay using MUO was used to quantify hGL activity. The assays were done in MUO substrate solution (10 mM acetate buffer pH 5, 0.15 M NaCl, 7 ppm Triton X-100, 0.15 mM MUO) as described before [18]. The assays were done in duplicates. The activity was expressed in µmoles of 4-methylumbelliferone (MU) produced per min per ml of undiluted extract or per mg of roots. It was then converted to units/mg of roots, knowing that one unit of enzyme catalyzed 0.219 µmoles of MUO per min under our assay conditions [18]. For the reaction on tributyrin, 100 µL of tributyrin was emulsified in 10 mL of 10 mM acetate buffer pH 5, 0.15 M NaCl, 1% molten agarose and the mixture was poured into a 90 mm diameter Petri dish. After setting, root powder was sprinkled on the plate. Photographs were taken after 1, 4, and 24 h.

### 2.4. Lipase Stability and Tube Binding Assays

Lipase was extracted from different batches of dry roots. Extracts were diluted 1/200 either in 50 mM glycine buffer at pH 2.2, 0.15 M NaCl or in 50 mM acetate buffer at pH 5, 0.15 M NaCl. The diluted extracts were kept in Protein LoBind^®^ Eppendorf tubes at room temperature and assayed for lipase activity after 1, 3, 6, 10, and 14 days. After 14 days, the solutions were transferred into new tubes and the lipase activity was assayed 24 h later. The activities in stored extracts were expressed as a percentage of activity related to freshly diluted extracts. To test for lipase binding to tubes, 100 µL of lipase diluted 1/200 in one or the other above buffer were placed in Protein LoBind^®^ Eppendorf tubes for 24 h. The solution was removed and the tubes were rinsed with 100 µL of buffer of the same composition as the one used to dilute the extracts. One hundred µL of MUO substrate solution was placed for one min in the empty tubes. One hundred µL of stop solution was added and the fluorescence of the MU (4-methylumbelliferone) was measured, indicating the activity of lipase bound to the tubes.

### 2.5. Cell Wall Polysaccharide Extraction

Alcohol-insoluble matter (AIM) was prepared using a method described in [20]. Two hundred mg of freeze-dried untransformed roots were ground in 2 mL of 70% ethanol. The suspension was vortexed for 20 s and centrifuged at 13,000× *g* for 30 s. The pellet was further extracted twice with 2 mL of 70% ethanol, twice with 100% ethanol, twice with chloroform/methanol (vol/vol), and once with acetone. The dry pellet was AIM. For pectin removal, AIM was extracted twice with 20 mM Tris-HCl pH 6.8, 50 mM EDTA for 15 min in an Eppendorf Thermomixer set at 60 °C and 1000 RPM, and then twice with 50 mM Na_2_CO_3_ under the same conditions. The pectin-deprived AIM pellet was then washed in 1 mL of acetone and dried at 65 °C.

### 2.6. In Vitro Lipase Binding Assays

Lipase was extracted from GL28 roots using LEB as described above (40 µL LEB/mg roots). The proteins were precipitated by 70% ammonium sulfate for 5 min at room temperature. After centrifugation for 5 min at 13,000× *g*, the pellets were dissolved in the same volume of 10 mM acetate buffer, pH 5. Two hundred µL of the solution was incubated for 15 min with 5 mg of either root powder or AIM or pectin-deprived AIM in an Eppendorf Thermomixer set at 30 °C and 900 RPM. The suspension was centrifuged at 13,000× *g* for 2 min. The supernatants were collected for the quantification of unbound lipase. The pellets were washed once with 10 mM acetate buffer pH 5 and resuspended in 200 µL of LEB for lipase extraction for 15 min in an Eppendorf Thermomixer set at 37 °C and 1000 RPM. After centrifugation at 13,000× *g* for 2 min, the lipase activity in the supernatant was assayed, indicating the amount of bound lipase.

### 2.7. Statistical Analysis

For each experiment, n indicates the number of lipase extractions done on dry roots. Two enzyme assays were done on each extract. The two values were averaged and the mean activities in the different extracts were calculated. The confidence intervals of the means (CI; *p* = 0.95) were calculated on vassarstats.net and indicated as error bars on the graphs. The number of replicates (*n*) is given for each experiment in the figures or after the mean values and CI in the text.

## 3. Results

### 3.1. Extraction of Lipase from Roots

**Figure 1:**
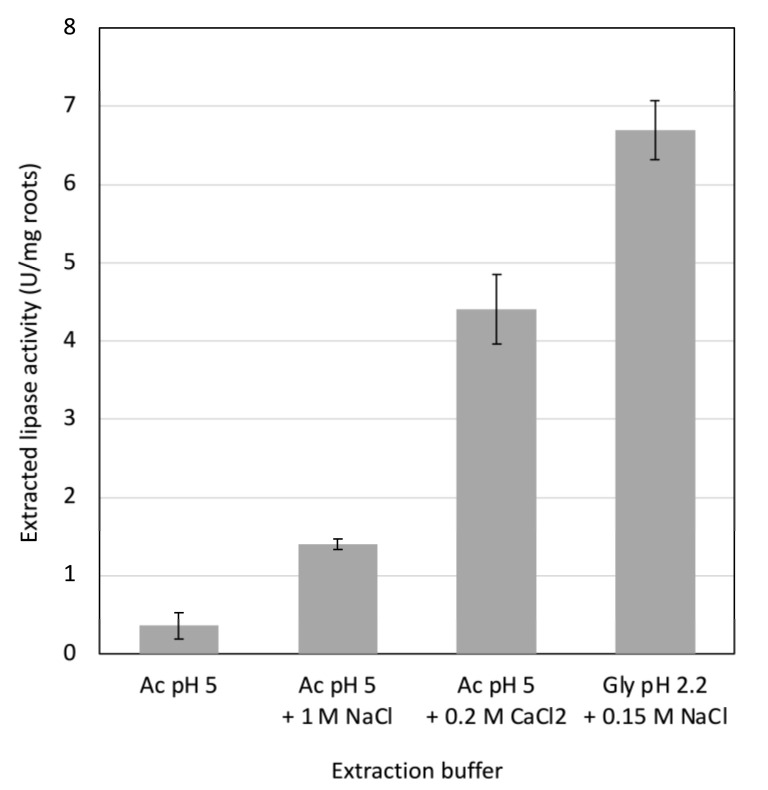
Release of gastric lipase from dry root powder in different solutions. Ac is 10 mM acetate buffer. Gly is 0.1 M glycine buffer. Bars are confidence intervals (0.95; *n* = 5).

Dehydrated roots were ground in various extraction buffers and the lipase activities in the extracts were measured. As found before [18], much less lipase was released in acetate buffer at pH 5 than in glycine buffer at pH 2.2 (LEB). However, the addition of 1 M NaCl or of 0.2 M CaCl_2_ to acetate buffer resulted in the release of 2.7 and 8 times more lipase activity than by acetate alone, respectively (Figure 1).

### 3.2. Stability of Lipase in Solution at Room Temperature

**Figure 2:**
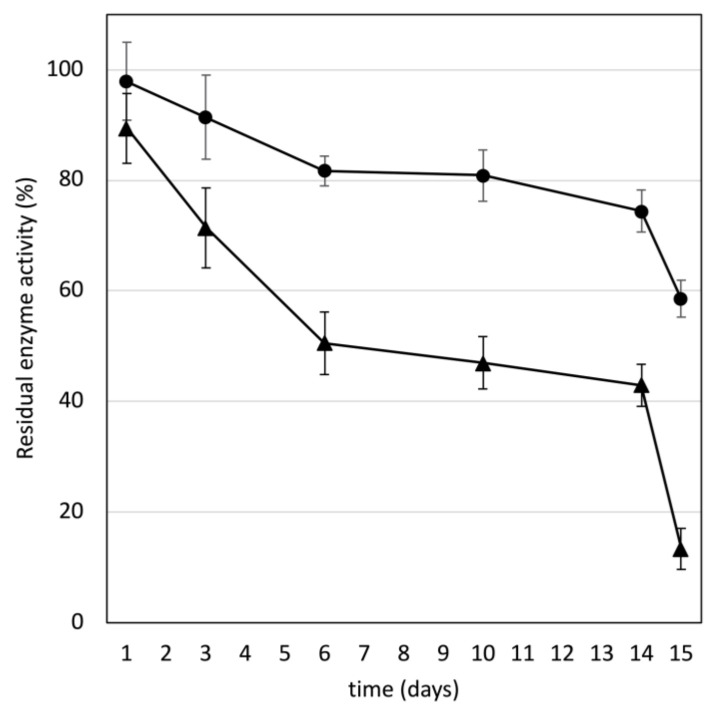
Time course of gastric lipase activity in extracts kept at 20 °C, at pH 2.2 (circles) or at pH 5 (triangles). The extracts were placed in new tubes on day 14. Bars are confidence intervals (0.95; *n* = 6).

A time course of lipase activity in solution at pH 2.2 or at pH 5 kept at room temperature was performed. These pHs were chosen because they are the pH of the extraction buffer and of the assay buffer, respectively. Whereas extracts at pH 2.2 retained approximately 75% of lipase activity after 14 days, those at pH 5 lost over 50% activity over the same time span (Figure 2). Transferring the diluted solutions of lipase to new tubes on day 14 resulted in a sharp drop of lipase activity after 24 h, especially at pH 5. To test for adsorption on tubes, lipase diluted in solutions at pH 2.2 or at pH 5 were placed in tubes for 24 h and removed. The tubes were then rinsed, and the activity of lipase adsorbed onto the tube was measured. The lipase activity per tube was 0.095 ± 0.012 mU (0.95 CI, *n* = 6) or 1.5 ± 0.39 mU (0.95 CI, *n* = 6) for lipase in buffer at pH 2.2 or at pH 5, respectively.

### 3.3. Long-Term Storage of Lipase in Roots

Extracts from roots that had been freeze-dried the day before had a lipase activity of 6.59 ± 0.26 U/mg roots (0.95 CI, *n* = 10). Extracts from roots that were kept in silica gel-containing pots for 27 months had a lipase activity of 6.63 ± 0.3 U/mg roots (0.95 CI, *n* = 10). Similar lipase activity was observed in extracts from dry roots that were stored for shorter amounts of time (Table S1).

### 3.4. Temperature Stability of Lipase

**Figure 3:**
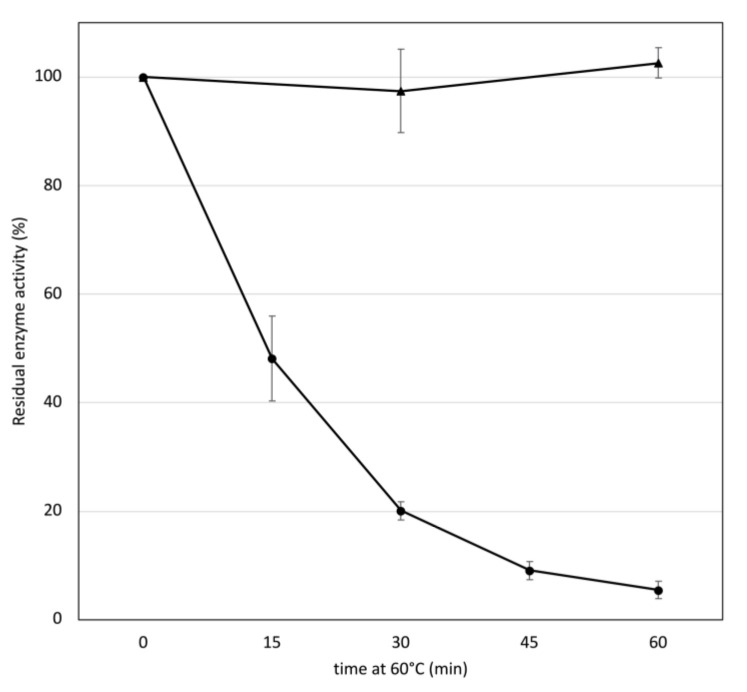
Stability at 60 °C of gastric lipase in solution in lipase extraction buffer (circles) or in dry roots (triangles). Bars are confidence intervals (0.95; *n* = 4).

To investigate whether the association of gastric lipase to roots translated into higher temperature stability, the stability at 60 °C of lipase in solution or in dehydrated roots was compared. The half-life of lipase in lipase extraction buffer at 60 °C was approximately 15 min whereas there was no loss of lipase activity extracted from dry roots kept at 60 °C for one hour (Figure 3). Incubating lipase in extraction buffer for 5 min at 80 °C resulted in a total loss of lipase activity (data not shown). In contrast, the activity of lipase extracted from dry roots incubated at 80 °C for one hour was 92.7 ± 9.4% (0.95 CI, *n* = 4) of that of lipase extracted from the control roots kept at room temperature. Similarly, 56.4 ± 17.1% (0.95 CI, *n* = 4) activity could be recovered from roots incubated for one hour at 100 °C. To assess the relative contributions of the association with roots or of the dehydration in the thermal stability of dry root-associated lipase, dehydrated roots were ground and hydrated in 20 mM acetate buffer pH 5, 0.15 M NaCl. The mixes were incubated either at room temperature or at 60 °C for 30 min. The lipase was then extracted from the roots and assayed for lipase activity. The activity extracted from heat-treated hydrated roots was 33.4 ± 5.4% (0.95 CI, *n* = 4) of the activity extracted from hydrated roots kept at room temperature.

### 3.5. In Vitro Binding Assays

**Figure 4:**
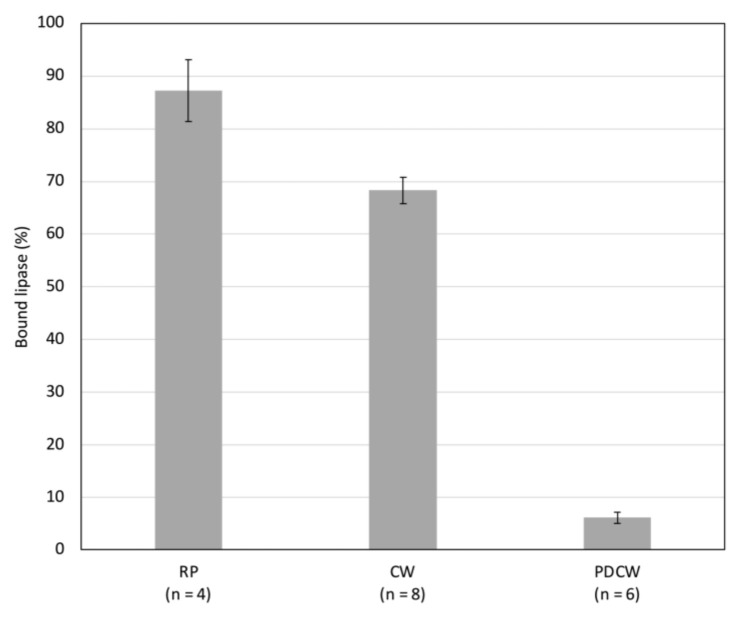
In vitro binding of gastric lipase to root powder (RP), cell wall powder (CW), or pectin-deprived cell wall powder (PDCW). Bars are confidence intervals (0.95, *n* indicated on graph).

To investigate the interaction of human gastric lipase with plant cell wall polysaccharides, in vitro binding assays were undertaken. Lipase in 10 mM acetate buffer at pH 5 was incubated with either untransformed root powder, cell wall powder, or cell wall powder from which pectin had been extracted. The unbound and bound fractions of lipase were extracted and assayed as indicated in the materials and methods section above. The binding of lipase to each powder was expressed as a percentage of bound lipase activity related to the total lipase activity. The binding of hGL was much more extensive on root powder and cell wall powder than on pectin-deprived cell wall powder (Figure 4).

### 3.6. Enzyme Release from Root Powder

**Figure 5:**
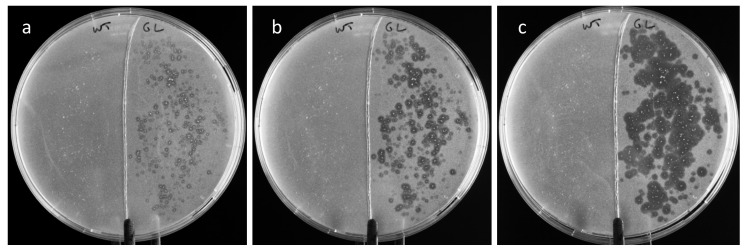
Degradation of tributyrin by gastric lipase diffusing from transgenic root powder on an agarose plate. (**a**) after 1 h; (**b**) after 4 h; (**c**) after 24 h. In each plate: left, untransformed root powder; right, transgenic root powder.

To see whether gastric lipase could be released from root powder to hydrolyze a substrate, root powder was sprinkled on a tributyrin-containing agarose plate. Untransformed root powder was used as a control. The formation of a clear halo around each grain of transgenic root indicated tributyrin hydrolysis, with the size of the halos increasing with time (Figure 5).

## 4. Discussion

Previous work has shown that unlike EGFP (enhanced green fluorescent protein), the human gastric lipase targeted for secretion failed to diffuse from transgenic hairy roots into the culture medium [18]. The enzyme, which had likely accumulated in the cell walls, had to be extracted from the roots. Similarly, gastric lipase produced in *Pichia pastoris* also remained in the cell wall [21]. The efficiency of the extraction of human gastric lipase from hairy roots was shown to be pH-dependent, with markedly more lipase being released at pH 2.2 than at pH 5 [18]. Here, the effect of salts on lipase release was investigated. The addition of 1 M NaCl or of 0.2 M CaCl_2_ enhanced the release of lipase from root powder at pH 5 (Figure 1). The effect of the 0.2 M CaCl_2_ solution on lipase release was found to be three times higher than that of the 1 M NaCl solution, although the ionic strength of the CaCl_2_ solution was lower than that of the NaCl solution. This result suggests a specific action of calcium ions. In plant cell walls, calcium ions interact with pectin by forming bridges between homogalacturonan chains [22]. The extensive effect of CaCl_2_ on lipase release from roots suggests involvement of pectin in lipase retention in the cell wall at pH 5.

To investigate the interaction of lipase with cell wall components, gastric lipase was added to either root powder or crude cell wall extracts or cell wall extracts from which soluble pectin had been removed. The binding of lipase to root powder and to cell wall powder was very effective whereas it was markedly lower on cell wall powder from which pectin had been extracted (Figure 4). This strengthened the hypothesis of lipase interaction with pectin in root cell walls. The negative charges of pectin at pH 5 might be responsible for the association with lipase, expected to be positively charged below its pI of 6.9. In contrast, at pH 2.2, homogalacturonans are expected not to be charged, which would explain the release of lipase at this pH. Similarly, the addition of CaCl_2_ might trigger lipase release at pH 5 by competing for the negative charges of pectin.

The time course analysis of lipase activity in solution at pH 2.2 revealed a slow decrease down to approximately 75% of the initial activity after 14 days (Figure 2). There was more activity loss at pH 5, down to approximately 45%. Although this could indicate higher stability of lipase at pH 2.2 than at pH 5, the following observation points towards another mechanism. The transfer of lipase solutions into new tubes after 14 days resulted in a sharp drop in activity after 24 h. This strongly suggested that lipase adsorption on tubes was occurring. This hypothesis was confirmed by a tube-binding assay that revealed lipase activity on the surface of the tubes. The binding was 15 times more extensive at pH 5 than at pH 2.2. The adsorption of various lipases on polypropylene beads has been reported, with the immobilization causing a loss of enzyme activity in some cases [23]. The propensity of gastric lipase to adsorb on surfaces has to be taken into account when using this enzyme.

Human gastric lipase was found to be more stable in gastric juice than when purified [24]. It was found that the half-life of purified gastric lipase was only 25 min at pH 2 and pH 3 and 101 min at pH 5 whereas it was over 24 h in gastric juice at pH 3 and pH 5 and 510 min at pH 2. The stability of recombinant lipase extracted from plant roots here was higher than that of native lipase in gastric fluid. Some plant co-extracted proteins or other molecules may have a stabilizing effect on lipase. Alternatively, higher stability might result from differences in the protein structures of the native and the recombinant enzymes, due to differences in the glycan structures deposited on proteins by animal and plant cells [16]. In any case, the higher stability of the recombinant enzyme extracted from plant roots is a valuable feature for its use as a biocatalyst.

The effect of the association of lipase with root cell wall on the lipase thermal tolerance was evaluated. The half-life of lipase in solution in LEB at 60 °C was approximately 15 min (Figure 3). In contrast, the half-life of lipase in dry roots at 100 °C was over an hour, revealing much higher temperature stability of the lipase in dehydrated roots than in solution. When hydrated roots were incubated at 60 °C for 30 min, the lipase activity dropped by 67% whereas lipase in solution under similar treatment lost 80% of its activity (Figure 3). The thermal tolerance of lipase in dehydrated roots is therefore mainly due to dehydration.

The protective effect of dehydration was also confirmed by long-term stability measurements. It has been previously shown that approximately 17% of lipase activity was lost in roots kept at room temperature for two months [18]. In that experiment, no silica gel was used for the storage of the roots, which resulted in the roots being exposed to atmospheric moisture. Here, the storage of roots in the presence of silica gel ensured more extensive dehydration and long-term preservation of lipase activity. There was no loss of extractable lipase activity in dehydrated roots over a period of 27 months. Although freeze-drying is a well-established method of tissue or protein preservation, long-term studies are lacking [25]. In order to maintain the activity of proteins upon freeze-drying, a protecting agent must often be added to them [26]. The high stability of human gastric lipase in lyophilized plant roots revealed a high tolerance of the enzyme to dehydration.

Tributyrin was hydrolyzed around root powder specked on an agarose plate (Figure 5), which indicated that some gastric lipase was released from the roots and diffused onto the agarose to hydrolyze the substrate. The slow release of lipase at pH 5 initiated the production of butyric acid, which decreased the pH around the roots, causing the release of more enzymes. This induction mechanism likely explains the diffusion of the enzyme on the agar plate, indicated by the enlargement of the halos around the specks, over 24 h.

## 5. Conclusions

This work has revealed interesting properties of a plant-produced human gastric lipase, such as high stability at room temperature and high tolerance to dehydration, that could be leveraged in the design of supplement formulations for the treatment of certain patients. The propensity of gastric lipase to bind pectin or to adsorb on surfaces suggests that the immobilization of the enzyme may be easy. The stability of the enzyme favors its use as a biocatalyst in solvents or in ionic liquids. The strong association of lipase with roots provided a slow-release form of the enzyme that can be utilized in biochemical reactions, whether in aqueous or non-aqueous solvents. The ease of production of this extremophilic human enzyme by plant roots enables the production of structural variants of the enzyme that will improve its properties and its biosynthesis capacity.

## Data Availability

Data is contained within the article or Supplementary Materials.

## Supplementary Materials

The following supporting information can be downloaded at: https://www.mdpi.com/article/10.3390/life12081249/s1, Table S1: Lipase activity extracted from roots kept at room temperature for various amounts of time.
