# Supplementary material for: Properties of Human Gastric Lipase Produced by Plant Roots
Source: Life (Basel). 2022 Aug 16;12(8):1249. doi: 10.3390/life12081249 (PMC9409913; doi:10.3390/life12081249)
Supplement: Supplementary file 1 [file life-12-01249-s001.zip › life-1842524-supplementary.pdf]

Table S1. Lipase activity extracted from roots kept at room temperature for various amounts of time. Activities are mean values ± confidence intervals (p = 0.95; n = 4).

Root storage time	Extracted lipase (U/mg roots)
1 day	6.4 ± 0.79
11 months	6.87 ± 0.84
19.5 months	6.98 ± 0.55
